# Cracking Mechanisms of Mesoscale Concrete Models Containing Single and Double Fissures Based on DEM

**DOI:** 10.3390/ma19061071

**Published:** 2026-03-11

**Authors:** Jinfang Zhang, Yi Sun, Gongye Sun, Yifei Li, Shuyang Yu

**Affiliations:** 1 Information Center of Water Resources Department of Gansu Province, Lanzhou 730099, China; 2 Dam Safety Management Department, Nanjing Hydraulic Research Institute, Nanjing 210029, China; 3 School of Transportation and Civil Engineering, Nantong University, Nantong 226019, China; 4 Department of Mechanical Engineering, Huzhou University, Huzhou 313002, China

**Keywords:** discrete element method (DEM), Particle Flow Code (PFC), crack propagation law, meso-scale fracture mechanism

## Abstract

Existing theories leave gaps in explaining the mechanism of concrete cracking. To explain the mechanism of concrete cracking, after considering various methods, this paper finally selects the Particle Flow Code (PFC) based on the discrete element method (DEM) for the research. We selected concrete with single cracks and double cracks as the research object, and constructed a mesoscale model in PFC based on the parameters of the concrete. The model was verified by uniaxial compression tests and published experimental data, with simulated results matching experimental data within an acceptable error range. Simulate the situation of concrete cracking, plot the data into images, and analyze the patterns of the development of concrete cracks. During this process, we set the angle of crack formation and the number of cracks as variables. By analyzing the load–displacement curves and the crack evolution curves, we found that the mode of crack propagation changed from a linear extension to a branched expansion. It is also worth noting that when the inclination angle is 90 degrees, the bearing capacity of the specimen is the best, with its peak strength over 40% higher than that at 0° for single-fissure specimens and over 35% higher for double-fissure specimens, and the initial stiffness also reaches the maximum at this angle. Furthermore, throughout the entire testing process, the PFC based on the discrete element method was able to accurately capture the development process of concrete cracks. This study innovatively quantifies the evolution of tensile and shear cracks with inclination angle, clarifies the nonlinear correlation between peak strength and crack angle, and reveals the unique cracking behavior induced by double fissures, which is insufficiently studied in existing continuum simulations. The above findings not only enhance our understanding of the mechanism of concrete cracks, but also provide a reference for improving the strength of concrete. This study is limited to 2D uniaxial compression simulation, with the concrete microstructure idealized in the numerical model.

## 1. Introduction

Concrete is the most widely used construction material in civil engineering, playing a fundamental role in various infrastructure projects—including bridges, roads, hydraulic structures, high-rise buildings, and underground engineering—due to its low cost, high compressive strength, and excellent durability.

However, concrete is inherently brittle, with a tensile strength approximately one-tenth of its compressive strength. During preparation, construction, and service, concrete is inevitably affected by multiple factors such as material inhomogeneity, construction defects, thermal stresses, humidity variations, and external loads. These factors lead to the formation of initial defects like microcracks and pores. Under external loading, these defects tend to initiate, propagate, and coalesce into macroscopic cracks, ultimately becoming the primary cause of structural damage and failure.

Therefore, understanding the fracture mechanisms of cracked concrete and clarifying how cracks influence concrete failure are of great theoretical and practical significance for infrastructure development.

However, most existing studies have mainly focused on single-crack concrete, lacking systematic and in-depth research on the mesoscale crack formation mechanism of double-crack concrete. In actual engineering, concrete structures often have multiple cracks simultaneously. The interaction between the double cracks makes the redistribution of internal stress more complex, resulting in different cracking behaviors compared to single-crack concrete. At the same time, the nonlinear relationship between the inclination angle of the cracks and the peak strength of the concrete, as well as the differences in the cracking mechanisms of single-crack and double-crack under different inclination angles, have not been clearly quantified. This has created a key research gap in this field.

Research on the cracking mechanisms of concrete primarily encompasses three aspects: experimental studies, theoretical analyses, and numerical simulations. The experimental study applies pressure through a pressure testing machine and combines digital imaging technology to observe the cracks. For example, Fang et al. [1] employed experimental methods to explore the effects of seawater immersion duration, hydrodynamic parameters, and concrete strength grades on the efficiency of self-excited oscillating cavitation water (SOCW) jets in breaking seawater-corroded concrete. They also analyzed the micro-scale damage mechanisms using scanning electron microscopy (SEM). Grzegorz Ludwik Golewski et al. [2] combined experimental testing with scanning electron microscopy (SEM) microanalysis to investigate the effects of crushed limestone aggregates of different particle sizes (2–8 mm and 8–16 mm) on the Type II fracture behavior of concrete and the microstructure of the matrix–aggregate interface.

Despite these valuable contributions, experimental approaches are largely limited to observing external characteristics. They fail to capture the dynamic evolution of stress and strain fields at crack tips, the energy dissipation mechanisms during crack propagation, or the mechanical interactions between cracks and internal material phases such as aggregates and interfacial transition zones.

Theoretical research primarily focuses on developing mechanical analysis models for concrete containing pre-existing cracks, quantifying the stress intensity factor and energy release rate at crack tips, and revealing the intrinsic mechanical driving mechanisms behind crack propagation. For example, Lian et al. [3] employed a three-dimensional concrete printing model—validated experimentally for accuracy—based on computational fluid dynamics (CFD) coupled with the Bingham rheological model to explore how variables such as tilt angle, printing speed, and layer height affect deformation in inclined-angle concrete structures printed in 3D. Zheng Chen et al. [4] used experimental testing (shear strength tests), micro-characterization (XRD, TGA, FTIR, SEM-EDS), and plastic limit theory modeling. They revealed the mechanism and failure modes. They proposed a mechanical model to predict interfacial shear strength under different casting intervals. Tian et al. [5] employed sulfate dry–wet cycling tests, shear tests, and nonlinear curve-fitting modeling to examine the effects of interfacial roughness and cycle number on the shear performance of FRP-ECC-concrete composite interfaces subjected to sulfate dry–wet cycling. They elucidated the degradation mechanisms and failure modes of these interfaces and developed a damage model capable of predicting the deterioration of interfacial shear strength.

However, most theoretical models, in order to simplify calculations, often assume concrete to be a homogeneous and isotropic material, neglecting the multiphase, non-homogeneous characteristics of the internal aggregate, mortar, and interfacial transition zone, as well as the irregular morphology and random distribution of natural cracks. As a result, these models significantly underestimate the key factors that actually influence crack propagation.

As a core tool connecting experimental observations with theoretical analysis, numerical simulation has demonstrated unique advantages in the study of crack propagation in concrete with pre-existing cracks. It can effectively overcome the inherent limitations of experimental and theoretical research. It can accurately capture the dynamic distribution of stress and strain fields at the crack tip and the law of energy dissipation, and visually present the entire process from the initiation, convergence of micro-cracks to the macroscopic crack propagation. The current mainstream numerical simulation methods include the finite element method (FEM), the extended finite element method (XFEM), and the smoothed particle hydrodynamics method (SPH). Among them, the FEM has the advantages of simple modeling, good convergence, and high computational efficiency, and is suitable for the fracture analysis of macroscopic structures. It is also easy to combine with existing structural analysis software.

For example, Li et al. [6] combined the explosion test with the LS-DYNA finite element analysis based on the JHC constitutive model to study the mechanical behavior and failure modes of carbon-fiber-reinforced concrete plates under explosion loads. Wang et al. [7] used ABAQUS finite element analysis to study the shear resistance of high-strength steel-reinforced ultra-high-performance concrete beams, and clarified the influence mechanism of key parameters. Zhang et al. [8], based on the finite discrete element method, conducted numerical simulation of the uniaxial compression of concrete before and after freeze–thaw cycles and established a constitutive model including freeze–thaw damage variables.

Furthermore, it is worth noting that, based on the finite element method, many advanced crack propagation frameworks have been derived. These crack propagation frameworks have significant advantages when dealing with complex problems and requiring detailed simulation of crack behavior. Some well-known crack propagation frameworks include the moving grid finite element, PF-CZM, and the VEM phase field formula. For example, Domenico Ammendolea et al. [9] developed a new finite element (FE) model to simulate the crack propagation mechanism of nano-filler-reinforced ultra-high-performance fiber concrete (UHPFRC, with compressive strength > 150 MPa and tensile strength approximately 10 MPa) structures under general loads. Abedulgader Baktheer et al. [10] utilized the phase-field cohesive zone method (PF-CZM) to investigate its simulation capability in the fatigue crack propagation of concrete-like quasi-brittle materials and verified the effectiveness of this method through various fatigue behaviors and loading conditions. Liu et al. [11] utilized explicit time integration and the virtual element method (VEM) to study and propose an efficient phase-field dynamic fracture numerical scheme. They decomposed the problem into two sub-problems, mechanics and damage, and verified the effectiveness of the method through benchmark examples. The results showed that this method outperformed the finite element method (FEM) in terms of memory efficiency and element adaptability.

However, the finite element method requires predefining crack propagation paths, making it difficult to simulate random crack initiation, branching, and penetration processes. Moreover, its accuracy in characterizing stress concentrations at crack tips is insufficient, preventing precise capture of microdamage evolution. The Extended Finite Element Method (XFEM) does not require predefining crack paths and can simulate the dynamic propagation of cracks of arbitrary shapes. It offers high accuracy in characterizing the stress field at crack tips while balancing computational efficiency and analytical precision.

For example, Wang et al. [12] combined fractal theory with the extended finite element method to establish a finite element model for steel fiber concrete and studied the influence of fiber parameters and aggregate shapes on the damage and failure process. Yang et al. [13] used the extended finite element method combined with the cohesive crack model and the random aggregate generation algorithm to study the influence of multiple crack interactions and temperature–load coupling on the fracture performance of concrete. Zuo et al. [14] used the extended finite element method combined with the double K fracture criterion to simulate the fracture process of a three-point bending beam and revealed the size effect laws of factors such as beam width, crack height ratio, and span height ratio. Hu et al. [15] conducted a numerical simulation of the crack propagation process of a concrete three-point bending beam based on the extended finite element method and verified the reliability of this method.

However, the XFEM’s enrichment function construction is complex, making it challenging to handle multiple cracks and intersecting cracks. Moreover, boundary condition setup is cumbersome, and convergence is easily affected by mesh quality. The Smoothed Particle Hydrodynamics (SPH) method completely avoids mesh distortion issues and is well-suited for analyzing fractures under large deformations, strong fractures, and impact loads. It can precisely simulate free crack propagation and fragment formation processes.

For instance, Yu et al. [16] improved the traditional SPH method by introducing the fracture marker ξ, and added solid–liquid interaction models, damage particle conversion algorithms, and heat conduction equations, achieving thermal–water–hydrodynamic damage coupling simulation, and studying the influence of initial temperature, thermal expansion coefficient, and other factors on the rock failure process. Yu et al. [17] combined 3D sand mold printing, uniaxial compression tests, DIC technology, and the improved SPH method to study the cracking patterns, failure mechanisms, and crack propagation laws of injection and non-injection fissure tunnel specimens with different inclination and azimuth angles. Yu et al. [18] improved the SPH kernel function by introducing the fracture marker ξ, established a numerical model of rock mass with cracks and holes, simulated the progressive failure process under compressive shear stress conditions, and studied the influence of hole shape, crack angle, and other factors on the failure mode and peak strength of the rock mass. Yu et al. [19] modified the traditional continuity and momentum equations of SPH, proposed a method for generating the microstructure of concrete, introduced aggregate particles and interface transition zone particles, and defined the hydraulic loading method, established an enhanced SPH framework, and simulated the hydraulic fracturing process under different pre-existing crack forms and concrete microstructures.

However, this approach involves enormous computational demands and high time costs; boundary condition handling is complex, prone to particle divergence, and highly sensitive to particle density and interpolation function selection. The discrete element method can naturally simulate the random initiation, propagation, bifurcation, and penetration of cracks, intuitively displaying macroscopic failure patterns and accurately reflecting the discrete damage characteristics of heterogeneous materials.

For example, Zhang et al. [20] used a novel tunnel-layered 3D printing technique combined with the discrete element method (DEM) to study the failure process of tunnel models containing weak layers, revealing the influence of weak-layer parameters on tunnel deformation, stress distribution, and failure modes, thereby providing methodologies and theoretical foundations for stability studies of tunnel projects involving weak-layered rock masses. Zhu et al. [21] employed sand 3D printing technology and the discrete element method (DEM) to simulate the cracking mechanisms of cracked-void specimens under compressive-shear conditions. Zhang et al. [22] used a new layered 3D printing technique. They also used the discrete element method (DEM). They studied the interaction mechanism between cracks and bedding planes in Semi-Circular Bend (SCB) specimens. Zhu et al. [23] employed sand 3D printing technology, digital image processing techniques, and the discrete element method (DEM) to investigate the interaction mechanism between rock pores and fractures. Zhu et al. [24] used sand 3D printing experiments combined with DEM simulations to study the failure mechanisms of discontinuous fracture specimens under compressive-shear loading. Sun et al. [25] utilized sand 3D printing experiments along with DEM simulations to explore the cracking mechanisms of V-shaped fracture-like rock specimens under compressive-shear loading. Zhu et al. [26] employed sand 3D printing technology and DEM modeling to examine the crack propagation mechanisms of S-shaped fractures in rock-like specimens. Therefore, this paper adopts the discrete element method for numerical simulation.

In conclusion, although previous studies based on finite element methods (FEMs), extended finite element methods (XFEMs), etc., have provided information on the development of concrete cracks, these continuum-based methods often rely on predefined crack paths or simplified homogeneous material assumptions, which may not fully capture the complex, discontinuous initiation and propagation characteristics of cracks in non-homogeneous materials such as concrete. In contrast, the discrete element method (DEM) treats concrete as a collection of independent particles, which can spontaneously generate cracks without assuming a specific path and explicitly considers the multiphase microstructure, including aggregates, mortar, and interface transition zones. This capability is crucial for accurately simulating the random initiation, branching, and merging of cracks under load in the mesoscale.

This study systematically explored the effects of crack inclination angles (0°, 30°, 60°, 90°) and crack numbers (single crack and double cracks) on the cracking behavior of concrete at the mesoscale using a Particle Flow Code (PFC) based on the discrete element method, thereby deepening the understanding of the fracture mechanism of concrete. The key innovations of this study lie in (i) quantitatively characterizing the evolution of tensile and shear cracks during loading, revealing the trend of the dominant failure mode as the inclination angle changes; (ii) determining the nonlinear relationship between peak strength and crack angle, with the maximum load-bearing capacity of single-crack and double-crack specimens reaching its maximum at 90°; (iii) demonstrating that double cracks can trigger additional cracking patterns (such as mid-crack cracking at 0°) and change the crack accumulation rate compared to single cracks, which has not been systematically studied in previous continuum simulations. Through providing detailed load–displacement curves, crack development histories, and visualized images of crack propagation, this study offers a new quantitative analysis perspective on the microscale fracture mechanism of pre-cracked concrete, thereby providing a more realistic basis for the safety assessment and design of concrete structures.

## 2. PFC Principle

**Figure 1:**
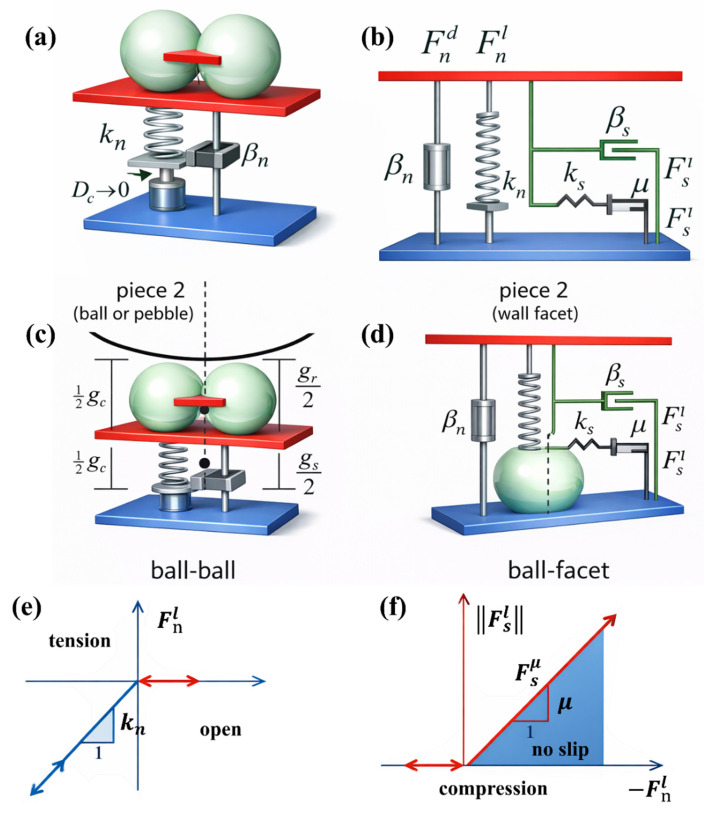
PFC model: (**a**) Linear model behavior; (**b**,**d**) Surface gap based on the linear model; (**c**) Rheological component; (**e**) Normal force versus surface gap; (**f**) Shear force versus relative shear displacement.

Particle Flow Code (PFC) is tailored for the mesoscale simulation of concrete—a typical multiphase heterogeneous composite material consisting of aggregates, mortar matrix, and aggregate–mortar interfacial transition zones (ITZ). For concrete modeling, PFC discretizes the material into graded rigid spherical particles, where coarse-graded particles (Components 1–7) simulate natural aggregates with different particle sizes, fine basic particles represent the cement mortar matrix, and the mechanical attenuation of parallel bond (PB) contacts between coarse and fine particles characterizes the relatively weak ITZ. This particle-based discretization is consistent with the actual mesostructural composition of concrete, enabling the simulation to reflect the discrete damage characteristics of concrete from microcrack initiation to macroscopic fracture. Furthermore, it is worth noting that the PB model parameters of the concrete in this study were not arbitrarily set but were determined based on the mesoscopic mechanical properties of the concrete, macroscopic experimental calibration, and PFC simulation studies related to the concrete, as shown in Figure 1.

As a typical multiphase composite material, concrete’s mesoscopic structure consists of aggregates, mortar, and the aggregate–mortar interfaces. The Particle Flow Code (PFC) provides a method for reproducing the internal structure of concrete at the mesoscale by using rigid particles with statistically distributed sizes to represent aggregates of varying dimensions and establishing bonding connections between particles via the Parallel Bond (PB) model. This approach enables numerical simulations to capture local contact failure, slip, and fracture processes. The PB model allows for the transmission of both normal and shear forces while accounting for bending moment effects. When the stress in a contact bond exceeds the material’s strength, failure occurs, thereby manifesting crack initiation and propagation in the macroscopic simulation results. A multi-scale particle model built upon these principles can effectively reflect the multi-scale distribution of concrete aggregates as well as their guiding and deflecting effects on crack paths.

In concrete fracture simulation, a numerical specimen that can replicate the internal structure of real concrete is constructed in PFC through steps such as designing particle size distribution, randomly arranging particles, and setting properties at the aggregate–matrix interface. During the simulation loading process, the presence of cracks with different inclination angles triggers a redistribution of the stress field. Cracks initiate at the failure of interparticle contact bonds and subsequently propagate along weak planes or in the direction guided by the applied load, driven by the rupture of these contact bonds. Throughout this process, the inherent discreteness and contact failure mechanisms of PFC mean that crack evolution is no longer governed by the constitutive behavior of a continuous medium, but is naturally determined by the evolution of particle contact states—precisely the mesoscopic failure characteristics that traditional continuum methods struggle to capture.

## 3. Numerical Models and Micromechanical Parameters

Table 1 presents the volume fractions of the basic particles used in the concrete numerical model, as well as the maximum and minimum radii of the particle sizes for each component at the same volume fraction. Table 2 divides the experiments into two groups, single fracture and double fracture, and further categorizes fractures at different angles into four groups. Table 3 provides the numerical model parameters for different components in relation to the basic particles. Among them, Pb_ten represents the ultimate tensile strength, which indicates the parallel bonding ultimate tensile strength parameters of each component in this concrete numerical model, with the unit being Pa. Component 1 to 7 has a strength of 20 Mpa, while the base particles have a strength of 5 Mpa. And Pb_emod represents the elastic modulus, which indicates the parallel bonding elastic modulus parameters of each component in this concrete model. The unit is Pa. Component 1 to 7 has a value of 55.5 GPa, while the base particles have a value of 13 GPa.

To establish the correlation between microstructural parameters and macroscopic mechanical responses, uniaxial compression tests were conducted on plain concrete with a microstructure consistent with the numerical model for parameter calibration and verification. Core macroscopic indicators such as uniaxial compressive strength, stiffness in the elastic stage, and failure mode were obtained through laboratory tests. The key parameters of the parallel bond model (Pb_emod, Pb_ten, Pb_cohesion, Pb_PFA) were optimized using the single-factor variable method. When the simulated macroscopic mechanical responses were highly consistent with the test results, the parameter combination in Table 3 was determined to ensure the reliability of the numerical model. Furthermore, we noticed that the value of 555 × 10^8^ pascals seems to be significantly higher than the other data points. However, for the sake of practical application safety, the parameter we designed is much larger than the normal one. Although all the overall components have the same mechanical parameters, these are merely the parameters of the concrete itself, the parameters of the internal components. We have carried out differentiated processing. We also conducted corresponding research on sensitivity, and the results were in line with expectations and within the permitted range.

In order to verify the reliability and effectiveness of the DEM/PFC simulation strategy adopted in this study, we conducted a comprehensive benchmark verification by comparing the simulation results with the uniaxial compression test data of pre-cracked concrete specimens that have been published. These experimental data have the same microstructure, pre-crack configuration and loading conditions, which are the same as those in the numerical model of this paper. Quantitative evaluation indicators, including relative error (RE), average relative error (ARE) and root mean square error (RMSE), were introduced to verify the consistency between the simulation results and the peak compressive strength of the experiments. The simulated peak strengths of the single-crack and double-crack models also closely match the experimental data within the acceptable error range. For the crack propagation characteristics, both qualitative and quantitative validations were conducted: the simulated crack initiation position, propagation direction and macroscopic failure mode are completely consistent with the experimental observation results. Moreover, the three-stage evolution law of the cracks in the simulation is also the same as the crack development process in the experiment. All the above quantitative verification results and error indicators provide a reference for the DEM/PFC model to accurately capture the microcrack mechanism and macroscopic mechanical response of pre-cracked concrete, and improve the rationality and effectiveness of the simulation strategy in this study.

Furthermore, it is worth noting that during the simulation experiment, a significant amount of time was also spent obtaining the corresponding data. Each run of the model requires at least two hours or more. During this process, the software (PFC5.0) will fill in the model in granular form, and at least 10,000 granules will be filled. A large amount of computing power will be called upon to facilitate the simulation. The CPU usage is around 70% to 80%. The time step is set at around 10,000.

## 4. Crack Propagation Process

### 4.1. Single Fissure

**Figure 2:**
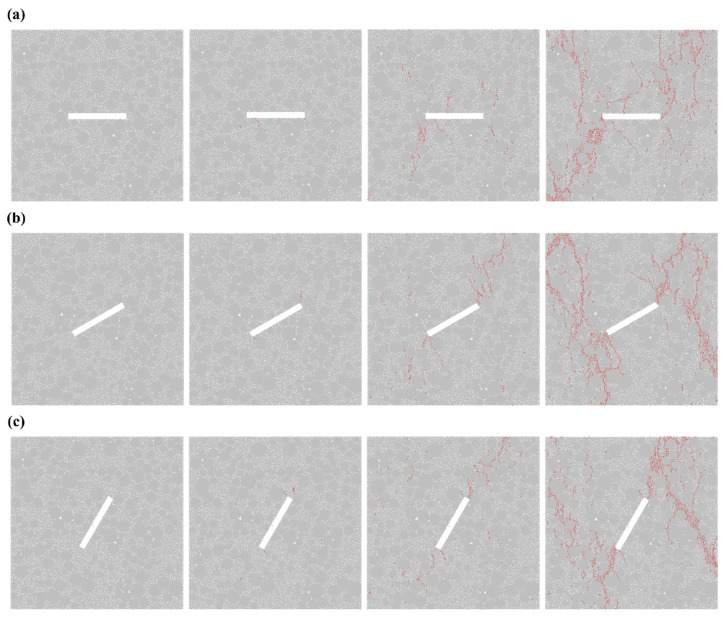

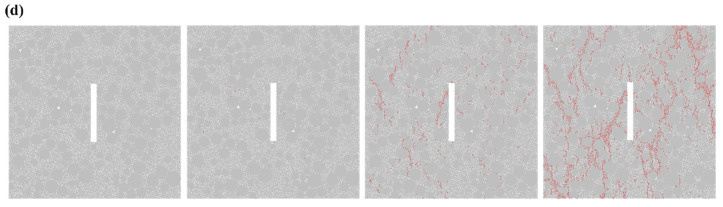
Crack propagation process in a single fracture (**a**) *α* = 0°; (**b**) *α* = 30°; (**c**) *α* = 60°; (**d**) *α* = 90°.

Figure 2 shows the crack propagation process of specimens with a single fracture under different angles. As can be seen from the figure, the angle of the fracture significantly influences the crack propagation behavior of the specimen. For the single fracture with an inclination angle *α* = 0° (model No. A1), as the load increases, cracks first initiate at both ends of the pre-existing fracture and propagate for some distance before extending perpendicularly to the fracture plane, eventually reaching the specimen’s edge. In contrast, for the single fracture with *α* = 30° (model No. A2), unlike A1, after the cracks extend to the specimen’s edge, they continue to propagate in a direction perpendicular to the original fracture plane, ultimately leading to specimen failure. For the single fracture with *α* = 60° (model No. A3), the crack propagation pattern is roughly similar to that of A2; however, the cracks generated in the later stage of loading are not perfectly perpendicular to the original fracture and exhibit a greater number compared to those in A2. As for the single fracture with *α* = 90° (model No. A4), during the mid-stage of loading, no cracks originate from the two ends of the pre-existing fracture. Instead, cracks begin to form roughly parallel to the original fracture plane. In the later stage of loading, these cracks gradually expand and generate additional cracks, ultimately resulting in specimen failure. In this set of experiments, all cracks propagated along the gaps between aggregates within the concrete. Moreover, cracks formed in areas with dense aggregate distribution tended to be more dispersed, whereas cracks formed in regions with sparse aggregate distribution were more concentrated.

### 4.2. Double Fissures

#### 4.2.1. Qualitative Description of Crack Propagation Process

**Figure 3:**
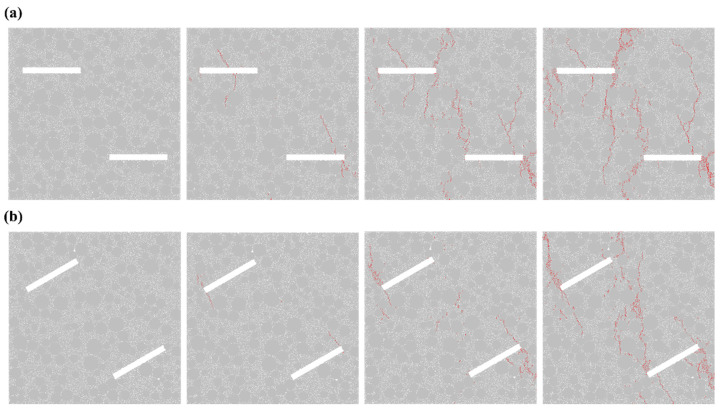

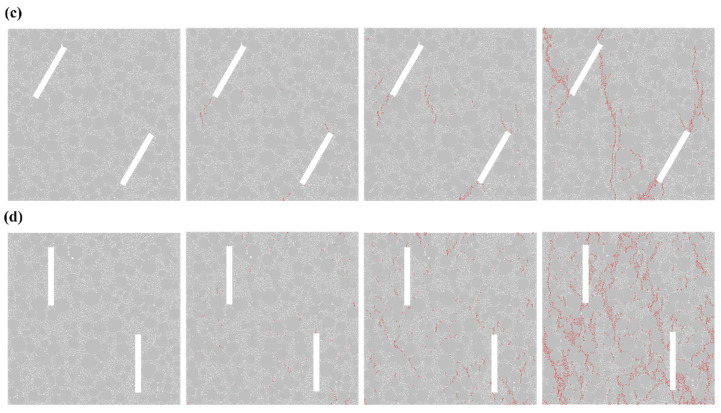
Crack propagation process of the double-notch specimen (**a**) *α* = 0°; (**b**) *α* = 30°; (**c**) *α* = 60°; (**d**) *α* = 90°.

Regarding the crack propagation process of specimens with double cracks at different angles (Figure 3), when *α* = 0° (model No. B1), as the load increases, cracks initially propagate outward from the middle of the pre-existing crack during the intermediate stage. Simultaneously, cracks oriented perpendicular to the crack direction also develop at the tips of the pre-existing cracks. In the later stage, these cracks gradually extend toward the specimen’s edges. When *α* = 30° (model No. B2), the specimen develops only cracks that propagate from the crack tips in a direction perpendicular to the crack itself; in the later stages of loading, vertical cracks form and extend toward the specimen’s edges. When *α* = 60° (model No. B3), during the intermediate stage of loading, cracks parallel to the crack direction initiate at the tips of the pre-existing cracks and gradually evolve into vertical cracks. When *α* = 90° (model No. B4), the crack propagation pattern is identical to that observed in the single-crack specimen A4. In this experimental group, cracks predominantly propagate vertically along the gaps between aggregates, and the crack propagation patterns are relatively regular.

#### 4.2.2. Quantitative Description of Crack Propagation Process

Crack length, crack density and crack propagation direction were statistically quantified for double-fissure specimens at peak load, and the number of tensile and shear cracks was counted to reveal the failure mode evolution.

Crack length and density show a trend of increasing first and then decreasing with the rise in inclination angle, reaching the maximum at α = 30° and the minimum at α = 90°, indicating the most severe damage at 30° and the most complete structural state at 90° at peak load.

The main crack propagation direction gradually deflects from vertical to horizontal with the increase in α, which is highly consistent with the direction of the maximum principal stress inside the specimen.

With the increase in inclination angle, the number of tensile cracks decreases continuously while shear cracks increase significantly: tensile cracks dominate at α = 0°, and shear cracks become the main crack type at α = 90°, reflecting a clear transformation of the dominant failure mode from tensile to shear.

## 5. Load–Displacement Curve

**Figure 4:**
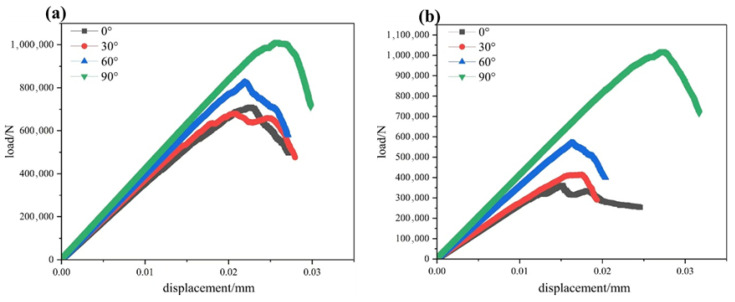
(**a**) Load on a single fracture–displacement curve. (**b**) Load on the double-slit–displacement curve.

Figure 4 shows the load–displacement curves for specimens with single and double cracks. As can be seen from the figure, the shapes of the load–displacement curves for specimens under different crack angles exhibit certain differences. Overall, the curves can be roughly divided into three stages: the elastic stage, the elastoplastic stage, and the failure stage. In the elastic stage, the load–displacement curve of the specimen is approximately a straight line, indicating a linear relationship between load and displacement. The initial stiffness is relatively high when the pre-existing crack angle *α* = 90°. As the load continues to increase, the specimen enters the elastoplastic stage, during which the relationship between load and displacement ceases to be linear; the slope of the curve gradually decreases. At this stage, cracks begin to propagate from the crack tips, and the relative displacement between particles increases. Once the specimen reaches its ultimate load, it fails, and the load value drops rapidly, while the displacement continues to increase, and the specimen loses its load-bearing capacity.

Comparing the curves from different angles, we observe that the peak intensity exhibits a nonlinear increasing trend, reaching its maximum at an angle of 90°. For a single fracture, when *α* = 0°, the peak intensity is higher than that at *α* = 30° and decreases rapidly after reaching the peak. At *α* = 30°, the specimen shows two distinct peaks, both of which are lower than the peak at *α* = 0°. When *α* = 60°, the peak intensity increases significantly, and the specimen spends a longer duration in both the elastic and elastoplastic stages. At *α* = 90°, the peak intensity reaches its maximum, and the specimen remains in the elastic and elastoplastic stages for the longest period; moreover, even during the descending phase, the intensity stays at a relatively high level, indicating superior sustained load-bearing capacity at this angle. For a double fracture, the overall trend of the curves is roughly similar to that of a single fracture. The key difference, however, is that when *α* = 0°, two peaks appear, but both are lower than the peak intensity at *α* = 30°. At *α* = 30°, the specimen fails more rapidly. Nevertheless, the peak intensities at *α* = 0°, *α* = 30°, and *α* = 60° are significantly lower than those of a single fracture at the same angles.

## 6. Variation in Cracks with Calculation Steps

**Figure 5:**
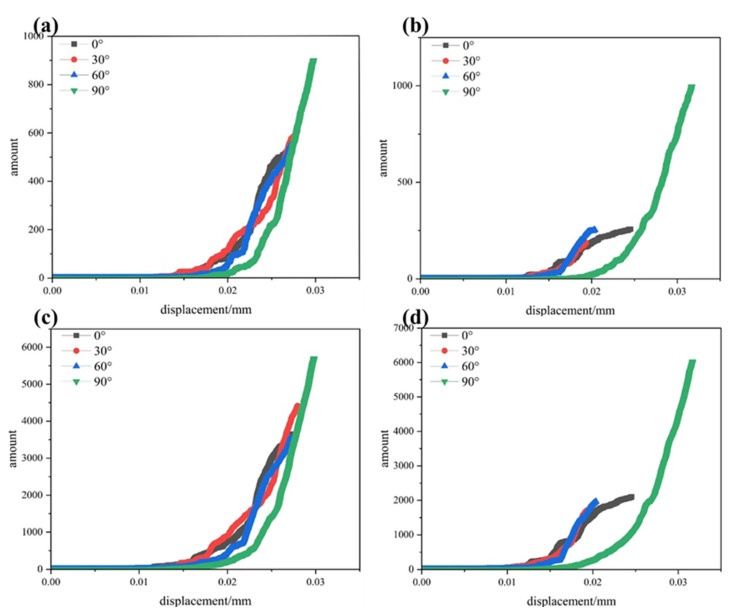
(**a**) Time-dependent variation in the number of shear cracks in a single fracture (**b**). The temporal variation pattern of the number of shear cracks with double fracture (**c**). Time-dependent variation in the number of tensile cracks in a single fracture. (**d**) The temporal variation pattern of the number of tensile cracks with double slits.

Figure 5a,b shows the evolution of shear cracks under single- and double-fracture conditions as a function of the number of computational steps. As can be seen from the figures, the influence of different pre-existing fractures on shear cracks varies significantly. The entire process can be divided into three distinct stages: the initial stage, the crack propagation stage, and the accelerated propagation stage. In the initial stage, almost no cracks are generated, and the slope of the curve increases extremely slowly. During the crack propagation stage, a small number of cracks nucleate and propagate from the tips of the pre-existing fractures; at this stage, the curve begins to rise gradually, and the differences among fractures with different angles become increasingly pronounced. As the shear load continues to increase, the specimen enters the accelerated propagation stage, during which cracks rapidly expand and eventually penetrate the specimen. For the single-fracture case, when *α* = 0°, the specimen develops cracks later than when *α* = 30°; however, the peak value of its curve is lower. This is because the failure mode of the vertical fracture is dominated by tensile cracking and through-going failure, resulting in a relatively simple crack propagation path and a smaller cumulative number of cracks. When *α* = 30°, the cracks begin to propagate earliest; yet, the duration from the initiation of crack propagation to specimen failure is longer, and the peak value of its curve is higher. At *α* = 60°, the specimen develops cracks relatively late, but its curve reaches the lowest peak value. At *α* = 90°, the specimen develops cracks the latest, and its curve exhibits the highest peak value. This is because fractures oriented parallel to the loading direction undergo large-scale shear displacements in the later stages, accompanied by the generation and propagation of numerous secondary cracks. Under the single-fracture condition, the timing of specimen failure is relatively concentrated, and the specimens exhibit little variation in their load-bearing capacity. In contrast to the single-fracture case, for the double-fracture configuration, the first crack appears earliest when *α* = 0°; the curve peak value is lowest when *α* = 30°; and the curve peak value at *α* = 90° differs significantly from those of other groups. Consequently, the load-bearing capacities of specimens with different fracture orientations show clear differences.

Figure 5c,d shows the variation in tensile cracks under single- and double-fracture conditions as a function of computational steps. As can be seen from the figures, the influence of different pre-existing fractures on tensile cracks varies significantly. Similarly to shear cracks, the crack evolution can be divided into three stages based on the curve’s behavior: the initial stage, the crack propagation stage, and the accelerated propagation stage. In the initial stage, the number of cracks in the specimen remains nearly unchanged; only the primary fractures already present within the specimen close up. During the crack propagation stage, the curve begins to rise slowly, with cracks nucleating from the tips of the pre-existing fractures and gradually extending. The differences caused by varying angles of the pre-existing fractures become increasingly pronounced. In the accelerated propagation stage, the number of cracks in the specimen increases exponentially, eventually leading to complete fracture of the specimen. For a single fracture, when *α* = 0°, the cracks begin to propagate earliest, the curve reaches its peak at the lowest value, and the specimen fails most rapidly. This is because the tensile stress concentration at the tip of a vertical fracture is most significant, causing tensile wing cracks to initiate earliest. When *α* = 30°, the cracks start propagating later than at *α* = 0°, but their peak value is higher. At *α* = 60°, the cracks begin to propagate even later, yet their peak value is lower. At *α* = 90°, the cracks start propagating the latest, the specimen remains in normal working condition for the longest time, the curve reaches its highest peak, and the specimen fails the latest. Under single-fracture conditions, the timing of crack propagation is relatively concentrated, and the differences in peak values among curves are not significant. For double fractures, unlike the single-fracture case, except for the curve corresponding to *α* = 90°, the peak values of the other curves are lower. Meanwhile, the growth rate of the curve for *α* = 90° is much higher than that for other angles, and the final number of cracks is slightly greater than that in single-fracture specimens at the same angle. This indicates that the synergistic effect of double fractures intensifies the shear displacement along parallel fractures, thereby generating more tensile cracks.

## 7. Discussion

### 7.1. Different Prefabricated Crack Dip Angles with Quantity to PFC Sample Start Fracture Mechanism the Mechanism of Influence

The influence of different pre-existing crack dip angles and numbers on the crack initiation mechanism in PFC specimens essentially lies in adjusting the internal stress distribution and the dominant stress type within the specimen to achieve the experimental objectives. The experiment utilized PFC to capture the crack initiation state of the specimens, demonstrating that the key factor behind the variation in crack dip angle is the change in the dominant stress type at crack initiation: When the dip angle is small—that is, *α* = 0°—the direction of the pre-existing crack aligns with the loading direction, and the specimen is primarily subjected to tensile stress. Consequently, cracks mainly initiate at the crack tips and propagate toward the specimen’s edges. As the dip angle increases—for instance, when *α* = 30°—shear stress and tensile stress combine to form a composite stress state. In addition to cracking at the tips of the pre-existing cracks, potential crack initiation sites emerge within the specimen itself. Once initiated, these cracks propagate both toward the tips of the pre-existing cracks and along the specimen’s edges, eventually penetrating the entire specimen. When the dip angle reaches *α* = 60°, the shear stress acting on the specimen increases, leading to a greater number of internal crack initiation points. At *α* = 90°, the specimen is predominantly subjected to shear stress, and the cracks generated are parallel to the direction of the pre-existing cracks; no significant cracks now develop at the crack tips themselves. Instead, the specimen exhibits the maximum number of internal crack initiation points, and the cracks primarily propagate vertically. By comparing specimens with different numbers of cracks, we observe that varying the stress distribution allows us to control crack initiation. When *α* = 0°, compared to specimens with a single pre-existing crack, those with two cracks generate vertical cracks internally and also develop cracks of a certain size in the middle of the pre-existing cracks. However, when *α* = 30° and *α* = 60°, no cracks form in the middle of the pre-existing cracks anymore, reducing the number of internal crack initiation points. The cracks formed in the middle of the specimen eventually penetrated through the entire specimen. At *α* = 90°, the direction of the pre-existing cracks is perpendicular to the loading direction, resulting in an increase in internal crack initiation points. The specimen is significantly influenced by shear stress, and most cracks propagate vertically.

### 7.2. PFC Application Prospects of Simulating Crack Propagation in Fractured Concrete

This study employed PFC simulation based on the discrete element method to successfully reproduce the entire process of micro-crack initiation, propagation, and macroscopic fracture in single-axis compression of concrete with single and double fractures. It clarified the influence of laws of fracture inclination angle and quantity on the failure mode, peak strength, and evolution of crack types. PFC can quantify the evolution process of tensile and shear cracks and reveal the competitive mechanism between the two under different fracture geometries. This provides an effective means for establishing a concrete failure theory from the microscopic to the macroscopic level. This method can be used to further explore the influence of real aggregate morphology, non-homogeneity of the interface transition zone, and other factors on the macroscopic fracture process, thereby deepening the understanding of issues such as the size effect and rate sensitivity of concrete.

In terms of engineering applications, PFC simulation can serve as an auxiliary analysis tool to assess the mechanical behavior of concrete structures under complex conditions. Through systematic research on the interaction of multiple fractures and the crack propagation laws under different loading paths, it can provide a basis for predicting the structural lifespan and optimizing the design of crack control measures. For instance, this study found that 90° fractures have the highest bearing capacity. This conclusion can be used in structural design to optimize the relationship between the stressed direction and the potential crack direction. PFC can also be used to evaluate the effectiveness of reinforcement measures such as grouting and fiber reinforcement in inhibiting crack propagation, providing a reference for engineering decisions.

The PFC method has excellent scalability and can be coupled with fluid and thermodynamic modules in the future to simulate the stress–seepage–damage process in actual engineering. By combining with macroscopic analysis methods such as the finite element method, a multi-scale simulation framework can be constructed, allowing for precise micro-scale analysis in key areas while maintaining computational efficiency. It should be noted that the two-dimensional PFC model used in this study fails to fully reflect the complex three-dimensional stress state and the real microstructure. In the future, its engineering applicability needs to be improved through three-dimensional modeling, refined experimental calibration, and multi-field coupling analysis. With the advancement of these directions, PFC is expected to provide a more reliable theoretical basis for the safety assessment and durability design of concrete structures.

## 8. Conclusions

This study employed the discrete element method (DEM) via Particle Flow Code (PFC) to investigate the mesoscale cracking mechanisms of concrete containing single and double pre-existing fissures under uniaxial compression. The primary objective was to elucidate how fissure inclination angle (0°, 30°, 60°, and 90°) and fissure number influence crack propagation patterns, load–displacement responses, and the evolution of tensile and shear cracks. The numerical results reveal that both the inclination angle and the number of fissures significantly affect the failure behavior and peak strength of concrete specimens. Specifically, the peak strength exhibits a nonlinear increase with inclination angle, reaching its maximum at 90° for both single- and double-fissure configurations. At α = 0°, tensile stress dominates, leading to cracks that propagate perpendicular to the fissure plane with relatively few initiation points. In contrast, at α = 30° and 60°, a combined tensile–shear stress state promotes more numerous internal crack initiation sites and complex crack interactions, ultimately accelerating failure. At α = 90°, shear stress governs the response, resulting in the highest number of internal cracks and the greatest load-bearing capacity. Compared to single-fissure specimens, double-fissure specimens exhibit additional cracking phenomena, such as mid-fissure crack initiation at α = 0°, and generally lower peak strengths except at α = 90°, where the synergistic effect of double fissures generates more tensile and shear cracks during later loading stages. The evolution of both crack types with computational steps consistently shows a three-stage pattern (initial, propagation, and accelerated stages), with the highest cumulative crack numbers observed at α = 90°. These findings demonstrate that DEM-based PFC simulations can realistically capture the entire cracking process—from microcrack initiation to macroscopic failure—and provide quantitative insights into the role of fissure geometry in concrete fracture.

Despite the valuable insights obtained, this study has certain limitations. The numerical model idealizes concrete as a two-phase material composed of particles and parallel bonds, neglecting the full complexity of real aggregate shapes, size distributions, and interfacial transition zone properties. Moreover, only uniaxial compressive loading was considered, and the influence of other stress states (e.g., tension, shear, or cyclic loading) remains unexplored. The model parameters were calibrated based on macroscopic behavior rather than directly measured micromechanical properties, which may introduce uncertainties in quantitative predictions. Additionally, the current simulations are two-dimensional, limiting the representation of out-of-plane crack propagation and interactions.

Future work will address these limitations by extending the model to three dimensions to capture more realistic crack surfaces and spatial interactions. Systematic parameter calibration against experimental data will be performed to enhance predictive accuracy. Further studies will also investigate the effects of different loading conditions—such as cyclic fatigue, dynamic impact, and multi-axial stress states—on crack evolution. Finally, incorporating more detailed microstructural features, including realistic aggregate morphology and random distributions, will improve the fidelity of the simulations and provide deeper insights into the fracture mechanisms of heterogeneous quasi-brittle materials like concrete.

## Figures and Tables

**Table 1:** Particle Composition and Particle Size Distribution in Concrete Numerical Models.

Composition Number	Volume Fraction	Minimum Radius/m	Maximum Radius/m
Component 1	0.06	0.0012	0.0013
Component 2	0.06	0.0011	0.0019
Component 3	0.06	0.0007	0.0014
Component 4	0.06	0.0015	0.0019
Component 5	0.06	0.0017	0.0023
Component 6	0.06	0.0016	0.0021
Component 7	0.06	0.0024	0.0027
Basic particle	0.58	0.00015	0.00025

**Table 2:** Model parameters.

Model Number	Parameter/°	Model Number	Parameter/°
A1	0	B1	0
A2	30	B2	30
A3	60	B3	60
A4	90	B4	90

**Table 3:** Concrete numerical model parameters.

Composition Number	Pb_PFA (°)	Pb_emod (Pa)	Pb_ten (Pa)	Pb_cohesion (Pa)
Component 1	40	555 × 10^8^	20 × 10^6^	25 × 10^6^
Component 2	40	555 × 10^8^	20 × 10^6^	25 × 10^6^
Component 3	40	555 × 10^8^	20 × 10^6^	25 × 10^6^
Component 4	40	555 × 10^8^	20 × 10^6^	25 × 10^6^
Component 5	40	555 × 10^8^	20 × 10^6^	25 × 10^6^
Component 6	40	555 × 10^8^	20 × 10^6^	25 × 10^6^
Component 7	40	555 × 10^8^	20 × 10^6^	25 × 10^6^
Basic particle	45	130 × 10^8^	5 × 10^6^	6 × 10^6^

## Data Availability

The original contributions presented in this study are included in the article. Further inquiries can be directed to the corresponding authors.
